# Probiogenomics Analysis of 97 *Lactobacillus* *crispatus* Strains as a Tool for the Identification of Promising Next-Generation Probiotics

**DOI:** 10.3390/microorganisms9010073

**Published:** 2020-12-30

**Authors:** Federico Fontana, Giulia Alessandri, Gabriele Andrea Lugli, Leonardo Mancabelli, Giulia Longhi, Rosaria Anzalone, Alice Viappiani, Marco Ventura, Francesca Turroni, Christian Milani

**Affiliations:** 1 Laboratory of Probiogenomics, Department of Chemistry, Life Sciences, and Environmental Sustainability, University of Parma, 43124 Parma, Italy; federico.fontana1@unipr.it (F.F.); giulia.alessandri@unipr.it (G.A.); gabrieleandrea.lugli@unipr.it (G.A.L.); leonardo.mancabelli@unipr.it (L.M.); giulia.longhi@unipr.it (G.L.); marco.ventura@unipr.it (M.V.); 2 GenProbio s.r.l., 43124 Parma, Italy; rosaria.anzalone@genprobio.com (R.A.); alice.viappiani@genprobio.com (A.V.); 3 Microbiome Research Hub, University of Parma, 43124 Parma, Italy

**Keywords:** comparative genomics, *Lactobacillus crispatus*, probiogenomics and vaginal microbiota

## Abstract

Members of the genus *Lactobacillus* represent the most common colonizers of the human vagina and are well-known for preserving vaginal health and contrasting the colonization of opportunistic pathogens. Remarkably, high abundance of *Lactobacillus crispatus* in the vaginal environment has been linked to vaginal health, leading to the widespread use of many *L. crispatus* strains as probiotics. Nevertheless, despite the scientific and industrial relevance of this species, a comprehensive investigation of the genomics of *L. crispatus* taxon is still missing. For this reason, we have performed a comparative genomics analysis of 97 *L. crispatus* strains, encompassing 16 strains sequenced in the framework of this study alongside 81 additional publicly available genome sequences. Thus, allowing the dissection of the *L.* *crispatus* pan-genome and core-genome followed by a comprehensive phylogenetic analysis based on the predicted core genes that revealed clustering based on ecological origin. Subsequently, a genomics-targeted approach, i.e., probiogenomics analysis, was applied for in-depth analysis of the eight *L. crispatus* strains of human origin sequenced in this study. In detail their genetic repertoire was screened for strain-specific genes responsible for phenotypic features that may guide the identification of optimal candidates for next-generation probiotics. The latter includes bacteriocin production, carbohydrates transport and metabolism, as well as a range of features that may be responsible for improved ecological fitness. In silico results regarding the genetic repertoire involved in carbohydrate metabolism were also validated by growth assays on a range of sugars, leading to the selection of putative novel probiotic strains.

## 1. Introduction

The host-associated microbiota is considered a primary contributor to the healthy status of many human compartments, such as the gastrointestinal tract (GIT) and the urogenital tract [1]. Regarding the latter, recent literature revealed that the vaginal microbiota is fundamental in both biological and chemical homeostasis [2]. In this context, it is well-known that bacteria harbored by the vaginal environment participate in maintaining a low pH, which on average should range between 3.8 and 4.5 in a healthy condition [3]. This supports the presence of a few dominant bacterial species resistant to low pH environments and concours in reducing the colonization by pathogenic species [3]. Among the dominant bacterial genera observed in the vaginal microbiota, the most prevalent are LAB bacteria, particularly the genus *Lactobacillus*, represented by the species *Lactobacillus iners*, *Lactobacillus crispatus*, *Lactobacillus jensenii,* and *Lactobacillus gasseri* [4]. Interestingly, in addition to the release of lactic acid and the acidification of the surrounding environment, these species have been shown to produce bacteriocins that counteract colonization of opportunistic pathogens [5].

An overview of the average vaginal microbiota composition in healthy women also revealed that the presence of specific dominant bacteria is associated with recurrent taxonomic profiles, known as community state types (CSTs) [6]. Five different CSTs have been identified in the literature, four of which (CST 1, 2, 3, and 5) showed a prevalent *Lactobacillus* composition, with the dominance of the species *L. crispatus*, *L. jensenii*, *L*. *gasseri,* and *L*. *iners*, respectively [6]. Remarkably, the CST 1, which is characterized by the dominance of the species *L. crispatus*, is known for its positive correlation with a healthy vaginal environment [6].

Nevertheless, the vaginal environment is often subject to dysbiosis, i.e., alteration of the taxonomic composition, due to the natural susceptibility of this environment to urogenital infections, or so-called bacterial vaginosis (BV). This condition is caused by opportunistic pathogens such as *Gardnerella vaginalis* or members of the *Prevotella* and *Atopobium* genera, which constitute the CST 4 [6]. Because of the relevance of *L. crispatus* in participating in vaginal health, many strains of this species have been industrially exploited as probiotics [7].

Concurrently, the species *L. crispatus* has been the subject of a broad body of literature focusing on dissecting their roles in human health through interaction with the host or competition with (opportunistic) pathogens, including interaction with VK2/E6E7 human vaginal epithelial cells and bacteriocin production [7,8,9,10]. Furthermore, in recent years, the degradative metabolism of *L. crispatus* toward glycogen, and the genetic differences between strains of human origin isolated from gut or vagina have also been investigated. The results of these studies highlighted some characteristics of *L. crispatus*, such as mechanisms of resistance and adaptation to the vaginal environment, leading to the growth of biotechnological and industrial interest toward the use of this species as a probiotic supplement [11,12].

Nevertheless, probiogenomics, i.e., the study of probiotic strains’ genetic repertoire, still have not been applied to identify putative novel *L. crispatus* of probiotic relevance through screening of human vagina isolates and their genetic comparison with publicly available *L. crispatus* genomes.

For this reason, in this study we performed an in-depth comparative genomic analysis between 97 different *L. crispatus* strains, including eight novel isolates from the vagina of healthy women and eight additional strains isolated from other hosts. Thus, leading to a thorough pan-genome analysis of *L. crispatus* complemented by a detailed functional dissection of the strain-specific genetic features. Such investigations were further extended by predicting bacteriocins-encoding genes and by an *in-silico* analysis of the glycobiomes of *L. crispatus* genomes that were validated by growth assays.

## 2. Materials and Methods

### 2.1. Genome Sequencing

For the purpose of this study, a total of 16 *L. crispatus* isolates were submitted to genome sequencing. DNA extracted from these isolates was subjected to whole-genome sequencing using MiSeq (San Diego, CA, USA) at GenProbio srl (Parma, Italy) according to the supplier’s protocol (San Diego, CA, USA). Fastq files of the paired-end reads obtained from targeted genome sequencing of isolated strains were utilized as input for genome assemblies through the MEGAnnotator pipeline [13]. SPAdes software was used for de novo assembly of each genome sequence [14], while protein-encoding ORFs were predicted using Prodigal [15].

Human participants gave their informed written consent before enrollment. All investigations were carried out following the principles of the Declaration of Helsinki.

### 2.2. Comparative Genomics

A pangenome calculation was performed using the pan-genome analysis pipeline PGAP [16], including each *L. crispatus* genome decoded in the framework of this study. Each predicted proteome of a given *L. crispatus* strain was screened for orthologues against the proteome of every collected *L. crispatus* strain by means of BLAST analysis (cutoff, E value of <1 × 10^−4^ and 50% identity over at least 80% of both protein sequences) [17]. The resulting output was then clustered into protein families by means of MCL (graph theory-based Markov clustering algorithm, using the gene family method [17]. A pangenome profile was built using all possible BLAST combinations for each genome being sequentially added. Using this approach, unique protein families encoded by the analyzed *L. crispatus* genomes were also identified. Protein families shared between analyzed genomes allowed us to identify the core genome of the *L. crispatus* species. Each set of orthologous proteins, belonging to the core genome, was aligned using Mafft software [18], and phylogenetic trees were constructed using ClustalW [19]. Based on these comparative analyses, a *L. crispatus* supertree was constructed and visualized using FigTree (http://tree.bio.ed.ac.uk/software/figtree/).

Using InterProscan [20], RapSearch2 [21] and NCBI CDD analysis [22], we also evaluated the presence of functional domains conserved within the unique genes present in the eight strains of *L. crispatus* isolated from human vagina present in our *L. crispatus* bacterial stock.

### 2.3. Carbohydrate Growth Assays

*Lactobacillus crispatus* strains were cultivated on semisynthetic MRS medium without glucose supplemented with a 1% (wt/vol) concentration of a particular sugar, and the optical densities (measured at a wavelength of 600 nm) were recorded using a plate reader (BioTek, Winooski, VT, USA). Tested sugars are arabinose, cellobiose, fructose, fucose, galactose, glycogen, glucose, inulin, maltodextrin, mannitol, N-acetil-D-glucosamine, N-acetil-D-galactosamine, mannose, melibiose, maltose, raffinose, rhamnose, ribose, sorbitol, starch, sucrose, threalose, turanose, and xylose (Figure 3). The plate reader was read in intermittent mode, with absorbance readings performed at 3-min intervals for three times after 24 h and 48 h of growth, where each reading was ahead of 30 s of shaking at medium speed. Cultures were grown in biologically independent triplicates, and the resulting growth data were expressed as the means of these replicates. Carbohydrates were purchased from Sigma and Carbosynth (Berkshire, UK). Carbohydrate-active enzymes were identified based on similarity to the carbohydrate-active enZYmes (CAZy) database entries [23].

### 2.4. Data Availability

The accession numbers of the genomic sequence of the 16 strains sequenced in this study are reported in Table S1.

## 3. Results and Discussion

### 3.1. General Genome Features of L. crispatus Strains Included in This Study

**Figure 1:**
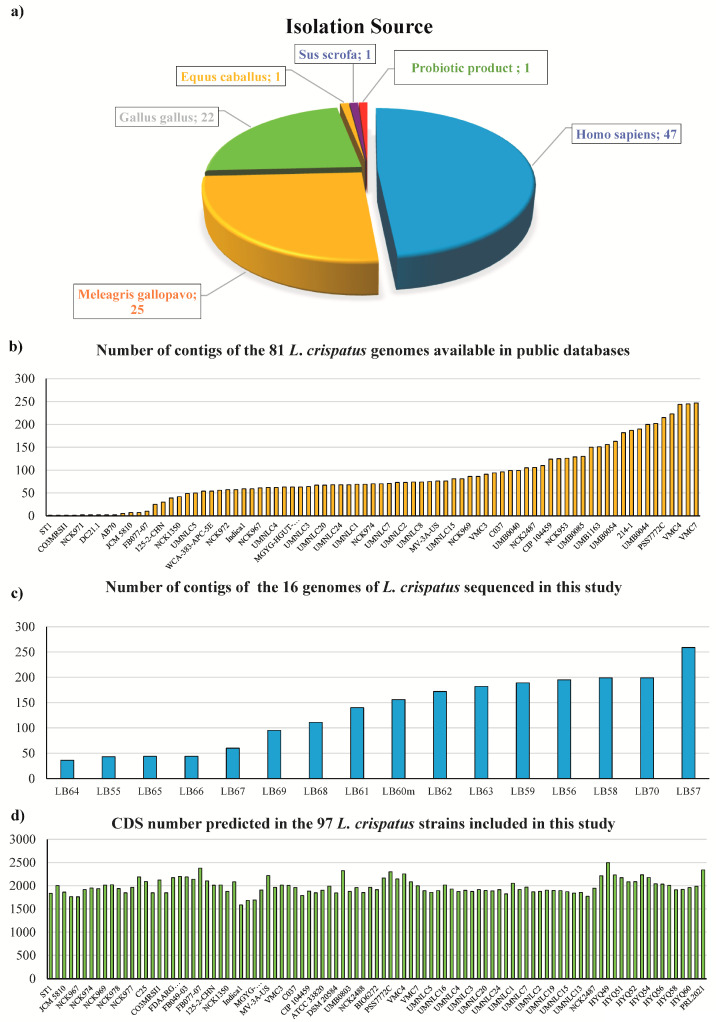
Graphic representation of the general genome features of strains included in the comparative genomics analysis. Panel (**a**) shows the subdivision of all the analyzed samples, through a pie chart, based on the isolation source. Panel (**b**) reports a bar plot illustrating the number of contigs of the *L. crispatus* genomes downloaded from public databases. Panel (**c**) shows a bar plot reporting the number of contigs of the 16 genomes of *L. crispatus* sequenced in this study. Panel (**d**) illustrate, by means of a bar plot, the number of CDS detected within each of the 97 strains of *L. crispatus* analyzed in this study.

A pool of 47 bacterial strains belonging to the *L. crispatus* species isolated from the human vaginal environment was used to perform a comparative genomics analysis. To dissect the specific genetic repertoire of strains isolated from the human vaginal tract, 50 additional *L. crispatus* strains isolated from other hosts, including *Sus scrofa*, *Equus caballus*, *Meleagris gallopavo* and *Gallus gallus*, were included in the analysis as an outgroup for comparative purposes (Table S1). As shown in Table S1, 16 of these strains were derived from our bacterial collection, of which eight were isolated from healthy women, one corresponding to a commercially available probiotic product, and seven isolated from poultry. Moreover, a total of 81 genomes were retrieved from those publicly deposited on NCBI after selecting those fragments in less than 250 contigs (Table S1, Figure 1). In addition, the average length of the genomes is 2120 Mb, with the smaller one with length of 1.641 Mb, thus ensuring adequate coverage with respect to the reference genome GCA_008694205.1, whose length consists of 2.2199 Mb (Table S1). Notably, poultry represents the most common ecological niche for publicly available genomes of *L. crispatus* along with human vagina (Table S1). In fact, 15 out of 81 genomes retrieved from NCBI are originated from the gut of chickens, 25 from the gut of turkeys, two derived respectively from pig and horse while 39 from the human vagina (Table S1, Figure 1). In order to obtain a comprehensive overview of the genetic variability of *L. crispatus* and identify the unique features of the strains of human origin, all the publicly available strains were included in this study.

The 16 *L. crispatus* strains whose genomes were decoded in this study resulted in an average of 84 contigs per genome, ranging between 36 and 259 contigs, as well as an average number of CDS equal to 2118, ranging between 1921 and 2496 (Table S1, Figure 1). Moreover, *L. crispatus* genomes retrieved from NCBI showed an average of 84 contigs per genome, ranging between one and 249 contigs, as well as an average number of CDS of 1957, ranging between 1585 and 2378.

### 3.2. Comparative Genomics Analysis of L. crispatus Strains

We performed a comparative genomics analysis through the use of the Pan-Genomes Analysis Pipeline (PGAP) software [16]. Clusters of Orthologous Groups (COGs) were defined as genes sharing >50% identity from alignments with >80% coverage. This analysis resulted in the prediction of the *L. crispatus* pan-genome, i.e., the entire set of genes belonging to this species and encoded by the strains analyzed, constituted by a total of 8387 COGs (Figures S1 and S2). Furthermore, the core-genome analysis allowed to identify 581 COGs shared by all genomes, thus representing the core-genome of the *L. crispatus* species. The 97 genomes included in this comparative genomics analysis were also screened for strain-specific unique genes. This analysis revealed that the pool of unique genes ranges from 11 to 137, with an average of 40 (Supplementary Excel File 1). In addition, we extrapolated the curves of both the pan-genome and the core-genome in order to evaluate their trend. The achieved pan-genome curve demonstrates that the large majority of the genetic diversity of *L. crispatus* is represented by the pool of sequenced genomes that we included in the analysis (Figure S2). Moreover, the *L. crispatus* core-genome appears to be stable at circa 750 COGs across the last five iterations.

**Figure 2:**
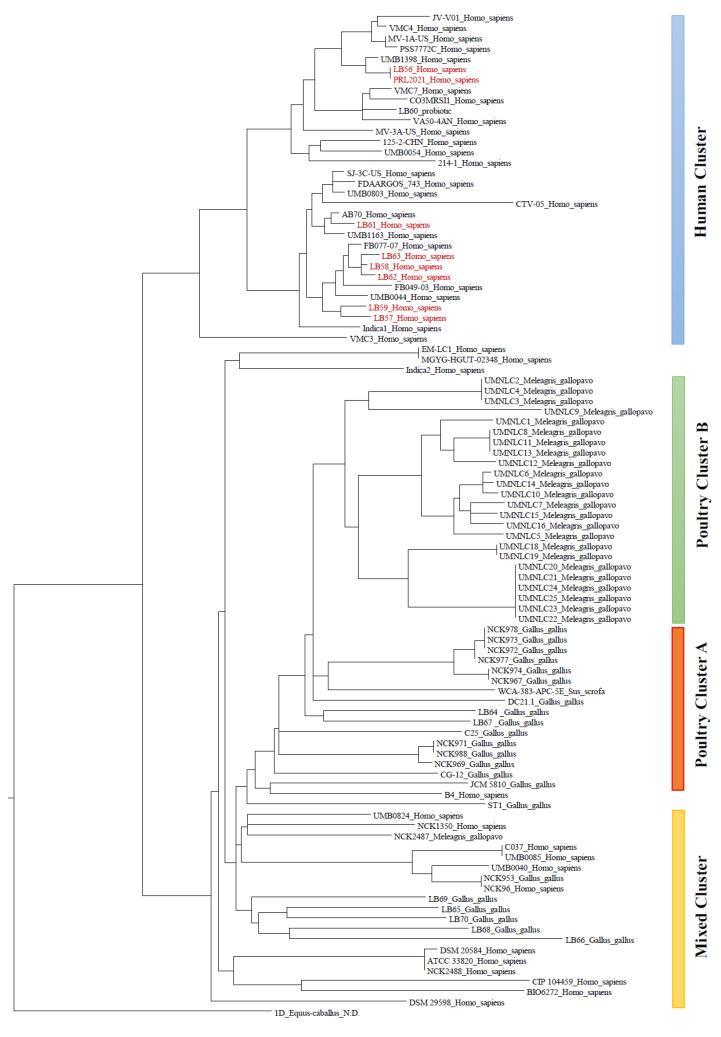
Phylogenetic core-genes tree of the 97 *L. crispatus* strains included in the comparative analysis. The figure shows the phylogenetic tree generated by alignment of the core genome previously detected among the 97 *L. crispatus* strains analyzed. The isolation source of each strain is also reported. Red text is used to highlight strains sequenced in this study and isolated from human.

Furthermore, we performed a phylogenomics analysis based on the alignment of the core gene sequences, which is an approach that has been shown to provide a detailed overview of the phylogenetic relationships between both distantly and closely related strains [24] (Figure 2). The reconstructed phylogenetic tree revealed clustering based on the strains’ ecological source, with a clear distinction between the genomes of human and animal origin (Figure 2). Interestingly, separate clusters were also observed between strains isolated from the poultry species *Gallus gallus* and *Meleagris gallopavo*, thus revealing peculiar host-specific adaptation of *L. crispatus* strains. In addition, a minor cluster with a mixed composition of strains of human and poultry origin was also identified, thus representing strains with putative multi-host specificity.

The phylogenetic tree obtained from the alignment of the *L. crispatus* core-genome allowed an in-depth exploration of the genetic features characterizing the strains of human origin (Figure 2). The 31 *L. crispatus* strains constituting the “Human” cluster observed in the phylogenetic tree (Figure 2) were used to identify the human-specific core-genome (HSCG), i.e., the set of COGs present in all human isolates and absent in the other genomes included in this study (Supplementary Excel File 1). Notably, no COG shared by all *L. crispatus* strains of human origin was observed. In fact, the analysis resulted in the identification of just 27 COGs shared by >50% of the 31 strains, of which the most shared COG was identified in only 84% (26/31) of the analyzed genomes. Thus, highlighting the high genomic variability of these strains despite their common ecological source, i.e., the human vagina, as also indicated by a higher average number of strain-specific (44) respect to that of the other genomes of strains isolated from non-human hosts (37) (Supplementary Excel File 1). Furthermore, we have screened the core-genome of strains isolated from chicken and turkey gut as for the strains derived from humans, i.e., focusing on the genes present only among the strains of a specific ecological niche. From this screening, we observed that the genomes of *L. crispatus* strains isolated from these two ecological niches are characterized by COGs shared by all the strains isolated from the same host. These results confirm the conclusions seen above for the human isolates, thus corroborating the high genetic variability of the whole *L. crispatus* species.

### 3.3. Comprehensive Genomic Characterization of L. crispatus Strains Isolated from the Human Vaginal Tract

*In silico* surveys exploiting a genomic approach, i.e., probiogenomics, is becoming crucial for the rapid screening of bacterial collections to identify optimal potential probiotic candidates, which will be further characterized by in vitro and in vivo experimental validations [25]. Following this approach, we performed a genomic dissection of the eight strains sequenced in the framework of this study that were previously isolated from the human vaginal environment of healthy women, in order to provide an example of how comparative genomics data can drive selection of new next-generation probiotics (Table S1).

Therefore, we analyzed the genomic repertoire occurring only and exclusively among the eight strains which were isolated from human sequenced in this study and not present in any other of the additional 89 *L. crispatus* strains left from the whole comparative genomics analysis, i.e., 81 publicly available genomes and eight strain of non-human origin sequenced in the framework of this project. Between the eight selected *L. crispatus* strains, an average of 53 genes (16%) were dispensable, a total of 278 (84%) were unique of a specific strain, ranging from 21 to 40, and no genes were shared exclusively between these eight strains (Figure S1, Table S2). Dispensable genes are those genes that are not included in the core-genome and that are also not unique genes of specific strains.

Subsequently, we performed a detailed analysis of the unique genetic repertoire of the eight selected strains by homology search in the NCBI RefSeq database and protein domain prediction based on a range of databases including PFAM, PANTHERA and CDD [22,26,27] (Table S2).

Of the 278 unique genes submitted to functional annotation, 168 resulted to encode hypothetical proteins, while among the 110 genes with a predicted function only 69 showed presence of a known protein domain. This functional prediction has been validated through the presence of specific functional domains, ranging from zero to 19 for each strain (Table S2, Figure 2). Moreover, of the 168 unique genes predicted to encode hypothetical proteins, only nine encompass a functional domain, seven of which correspond to the Domain of Unknown Function DUF4044-type domain (Table S2). Intriguingly, *L. crispatus* LB57 genome displays the highest number of unique genes, i.e., 49, of which 19 showing conserved functional domains, followed by the genomes of LB58 and LB56 with 48 and 40 unique genes, respectively (Table S2, Figure 2). Furthermore, the genome of LB57 presents intriguingly unique genes, such as transporters for sugars (HYQ49_1573 and HYQ49_2460), a transporter putatively involved in exporting the thiol-containing redox-active molecules cysteine and glutathione (HYQ49_1855 and HYQ49_1856) [28], two flippases (HYQ49_1948 and HYQ49_2461) and a heavy metal resistance efflux pump (HYQ49_2249) as indicated by substrate specificity prediction by homology search in the Transporter Classification DataBase (TCDB) [29] (Table S2). Moreover, LB57 showed the presence of genes encoding for putative surface proteins that may be involved in the interaction with the surrounding environment (HYQ49_2454, HYQ49_2521, HYQ49_2583 and HYQ49_2591), a gene participating in D-alanyl-lipoteichoic acid biosynthesis (HYQ49_2170) and a range of peptidases (HYQ49_0166, HYQ49_2407, HYQ49_2520, and HYQ49_2586) (Table S2). LB56 and LB58 strains also showed interesting unique genetic features. The unique gene set of LB56 is characterized by the presence of a type II toxin-antitoxin system (HYQ48_2254 and HYQ48_2255), a PTS transporter for sugars (HYQ48_1404 and HYQ48_1405) and a nitroreductase family protein (HYQ48_0882), while LB58 encompass a putative listeriolysin S family TOMM bacteriocin (HYQ50_0043), a type II toxin-antitoxin system (HYQ50_2190) and three putative cell wall-anchored proteins (HYQ50_2017, HYQ50_2201, and HYQ50_2202) (Table S2). Nevertheless, further functional analyses, e.g., transcriptomics and proteomics experiments, will be needed to corroborate these in silico analyses.

### 3.4. Prediction of Bacteriocin Production by L. crispatus Strains

Bacteriocins are peculiar proteins, produced by some bacterial strains, able to selectively inhibit the growth of a very limited range of bacterial targets [30]. In nature, they are produced by specific bacterial strains providing an ecological advantage in their niches toward specific competing strains [30]. Therefore, it is of high probiotic interest to study the presence of genes related to the synthesis of bacteriocins, as they can be fundamental in defining potential superior colonization capabilities of a specific strain or in limiting the occurrence of pathogens.

**Figure 3:**
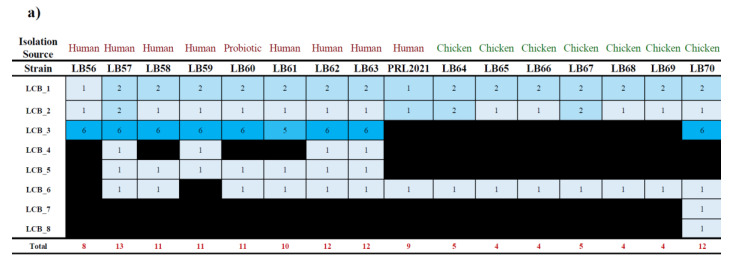

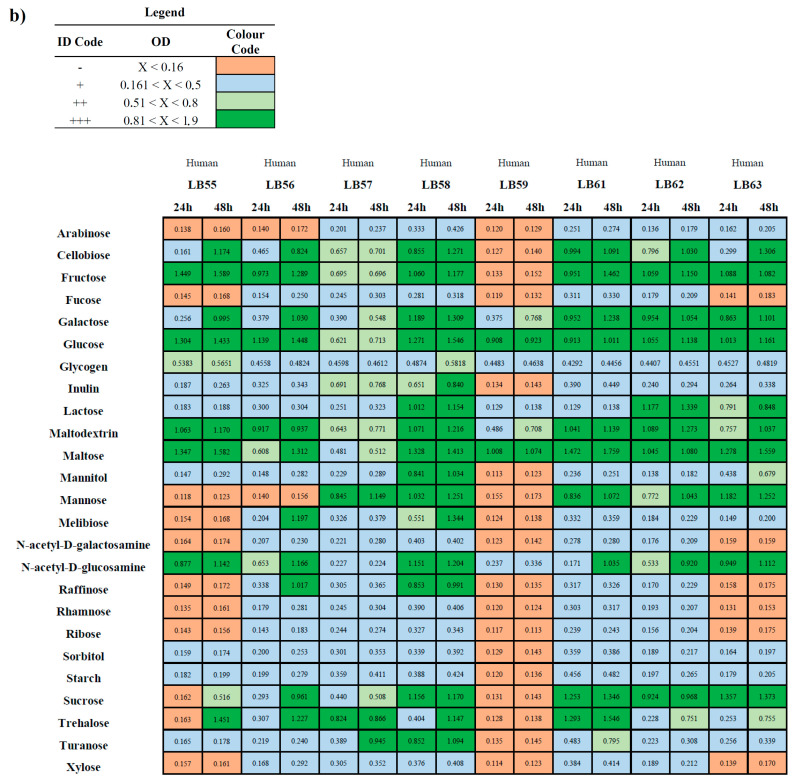
Analysis of the distribution of genes for bacteriocins and growths on specific sugars. Panel (**a**) shows a table illustrating the distribution of genes involved in the synthesis of bacteriocins among the 16 strains *of L. crispatus* sequenced in this study, with subdivision by isolation source. Additionally, a color coding was used to further highlight the distribution of these genes, with blue indicating high presence and black indicating complete absence of the genes. Panel (**b**) reported the growth performances of the eight strains of vaginal origin sequenced in this study when cultivated on media enriched with a range of different carbon sources.

We performed an analysis of the genes related to bacteriocin production using BAGEL4 tool on the 16 strains we isolated and sequenced [31] (Table S3, Figure 3). Through this analysis, we could identify a total of six putative bacteriocins genes/loci, named LCB (Lactobacillus Crispatus Bacteriocins) 1–6, distributed among the eight *L. crispatus* isolates of human origin and additional two in the strains isolated from poultry (LCB 7–8). Intriguingly, LCB 3 is constituted by a locus of six genes that include a predicted two-component regulatory system, a putative dedicated ABC transporter, a small peptide pheromone along with an immunity protein. This locus is absent in the isolates from poultry, while it is present in all the eight *L. crispatus* strains isolated from humans. In-depth analysis of this bacteriocin family based on PFAM data (https://www.ebi.ac.uk/interpro/entry/pfam/PF10439/) revealed that it may be implicated in inhibiting the growth of streptococci and closely related taxa [32].

Moreover, the genome analysis of the *L. crispatus* strains isolated from healthy human vagina revealed that LB57 is the one with the highest number of genes for bacteriocins synthesis, i.e., 13 genes, encoding for LCBs 1, 2, 3, 4, 5, and 6 (Table S3, Figure 3). Thus, representing the *L. crispatus* strain with the broader genetic potential in bacteriocin production. Furthermore, the *L. crispatus* LB62 and LB63 strains also showed the presence of LCBs 1, 2, 3, 4, 5, and 6, thus highlighting their potential interest for industrial applications, followed by LB58, LB59, LB60, and LB61 showing a total of five LCBs. In contrast, LB56 showed the presence of just three LCBs (Table S3).

### 3.5. Glycobiome Prediction of L. crispatus and Experimental Validations

The glycobiome is defined as the genetic repertoire related to sugar metabolism responsible for the simple and complex carbohydrate utilization by bacteria [33,34]. Based on this genetic background, each bacterial strain may exhibit unique growth characteristics in environments with specific carbohydrates availability, providing them specific ecological advantages when competing with other strains. Therefore, the prediction of the glycobiome in putative probiotic bacteria is of fundamental importance to define the possible colonization and persistence capabilities of a strain in relation to the availability of natural carbon sources in the environment [35].

**Figure 4:**
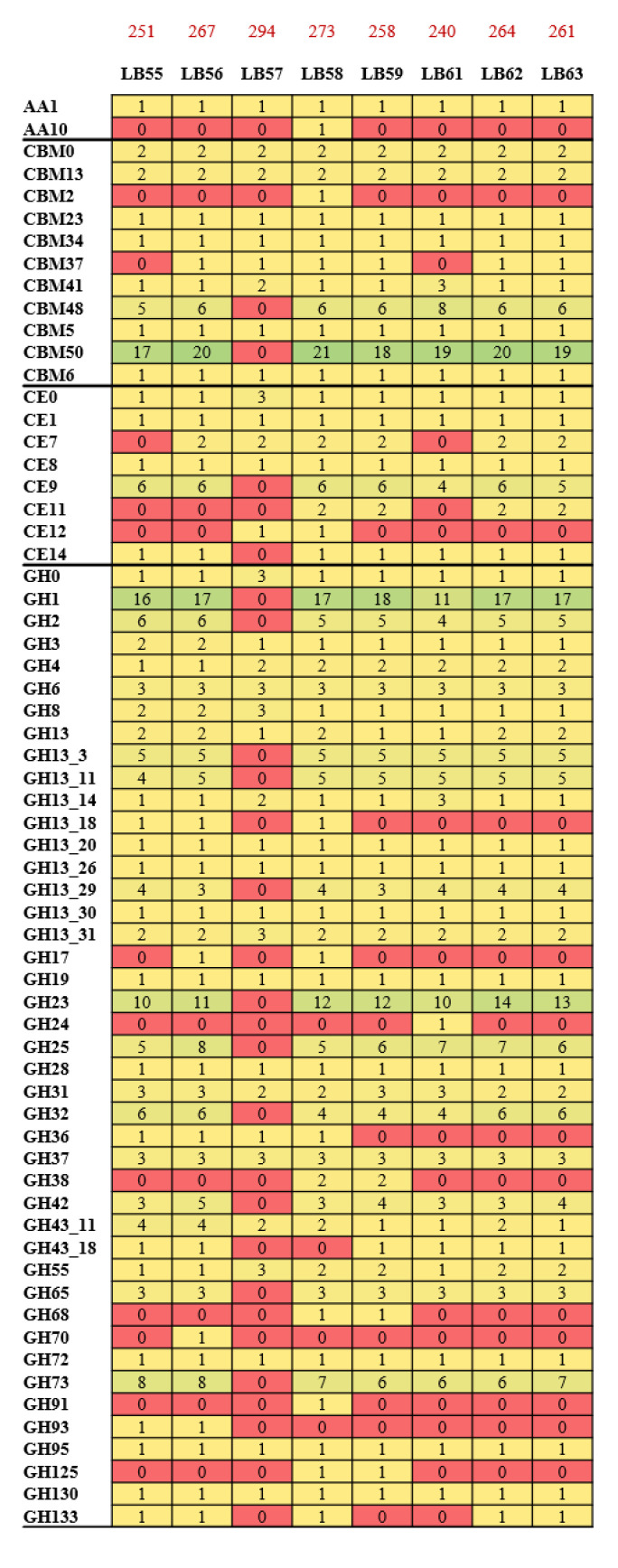

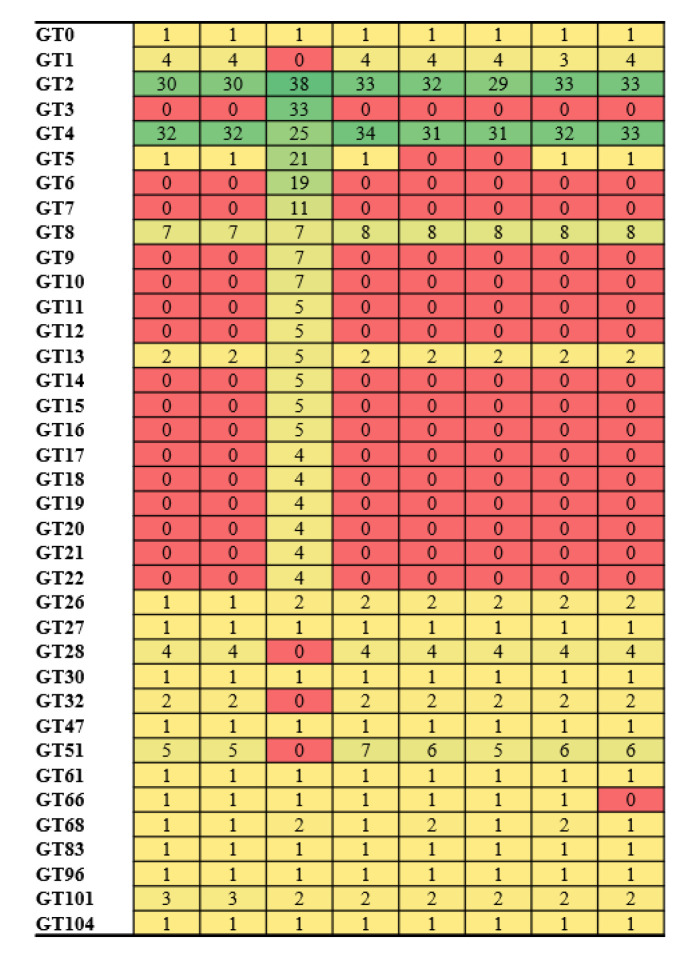
Predicted glycobiome of the eight strains of *L. crispatus* isolated from human vaginal sequenced in this study. The heat map shows the number of genes inherent to that specific class of the glycobiome, i.e., glycosyl hydrolases (GH), glycosyl transferases (GT), carbohydrate esterases (CE), carbohydrate binding module (CBM), or auxiliary activities (AA). The data was colored incrementally, starting from red to address the absence of related genes, up to green in cases where the number of hits, and therefore of genes, is high, with the maximum reported value being 38.

So, we analyzed the glycobiome profiles of the eight *L. crispatus* strains of human origin sequenced in this study in order to obtain a comprehensive overview of their ability to metabolize different sugars (Figure 4). Notably, the results showed similar glycobiome profiles among the analyzed strains, with the only exception of the predicted glycobiome of *L. crispatus* LB57. (Figure 4). In fact, this strain showed the higher abundance of genes constituting the glycobiome, i.e., 294, followed by LB58 and LB56 with 273 and 267 genes, respectively. Remarkably, LB57 also showed the absence of genes encoding for 11 glycosyl hydrolases (GH) and four glycosyl transferases (GT) that are instead present in all other strains and displayed a concomitant high abundance of genes classified as a range of 16 GT families absent in the other genomes (Figure 4). Furthermore, the genome of LB57 encodes 33 genes classified as GT3, absent in the other isolates, involved in glycogen synthesis (Figure 4). This may indicate that this strain has evolved to use glycogen for energy reserve, as previously observed in other bacteria, resulting in higher resistance to starvation and improved ecological fitness [12,36]. GT3 genes have been previously observed in *L. crispatus* strains isolated from vaginal microbial populations characterized by lactobacilli dominance [12]. Notably, their presence in *L. crispatus* genomes has also been linked to the synthesis of cell surface glycoconjugates that may be implicated in interactions with the host’s epithelial cells [12].

To validate these in silico data, we performed fermentation profiling analyses of these *L. crispatus* strains cultivated on 23 different sugars as the only carbon source, aiming to cross these data with the predicted glycobiome results (Supplementary Excel File 1, Figure 3). Notably, the cultivation of these strains on 25 carbohydrates, including both simple and complex sugars, corroborated the in silico glycobiome data (Figure 3). Remarkably, among the *L. crispatus* strain isolated from the vaginal environment, LB57 showed a peculiar growth profile, thus reflecting its glycobiome profile (Figure 3 and Figure 4). Nevertheless, growth on glycogen resulted in performances of LB57 similar to the other strains, suggesting that the high number of GT3 genes identified by glycobiome analysis may not be involved in glycogen degradation and may participate in other biological processes such as the synthesis of cell surface glycoconjugates [12].

## 4. Conclusions

We performed a comprehensive comparative genomics analysis of 97 strains of *L. crispatus* encompassing 81 publicly available genomes and 16 strain sequenced in the framework of this study resulting in the reconstruction of the pan-genome of this species. Data collected were used to define the core-genome of *L. crispatus* and to perform an in-depth phylogenomics analysis. Thus, revealing that strains isolated from the human vagina are characterized by high genetic variability despite their close phylogenetic relationship. Moreover, a probiogenomics approach was used to investigate the genetic potential of the eight *L. crispatus* strains isolated from humans and sequenced in this study in order to identify putative novel probiotics for the treatment of vaginal dysbiosis. Interestingly, unique genes putatively involved in improved colonization capabilities and (opportunistic) pathogens competition have been detected. In detail, the strain LB57 showed the broader repertoire of unique strain-specific genes as well as of genes for the synthesis of bacteriocins along with a peculiar carbohydrate metabolism that was validated by growth assay. Notably, this strain also showed a high abundance of glycosyl transferases of class 3, which may be involved in glycogen metabolism and in the synthesis of cell surface glycoconjugates that may be implicated in interactions with the host’s epithelial cells. Furthermore, while LB56 and LB58 also represented optimal candidates for further investigations due to their peculiar unique features involved in energy harvesting and host-microbe interaction, LB58 showed a broader repertoire of genes involved in the synthesis of bacteriocins that may provide superior ecological fitness.

## Data Availability

The accession numbers of the genomic sequence of the 16 strains sequenced in this study are reported in Table S1.

## Supplementary Materials

The following are available online at https://www.mdpi.com/2076-2607/9/1/73/s1.
