# Supplementary material for: Probiogenomics Analysis of 97 Lactobacillus crispatus Strains as a Tool for the Identification of Promising Next-Generation Probiotics
Source: Microorganisms. 2020 Dec 30;9(1):73. doi: 10.3390/microorganisms9010073 (PMC7824148; doi:10.3390/microorganisms9010073)
Supplement: Supplementary file 1 [file microorganisms-09-00073-s001.zip › Figure_S1.pdf]

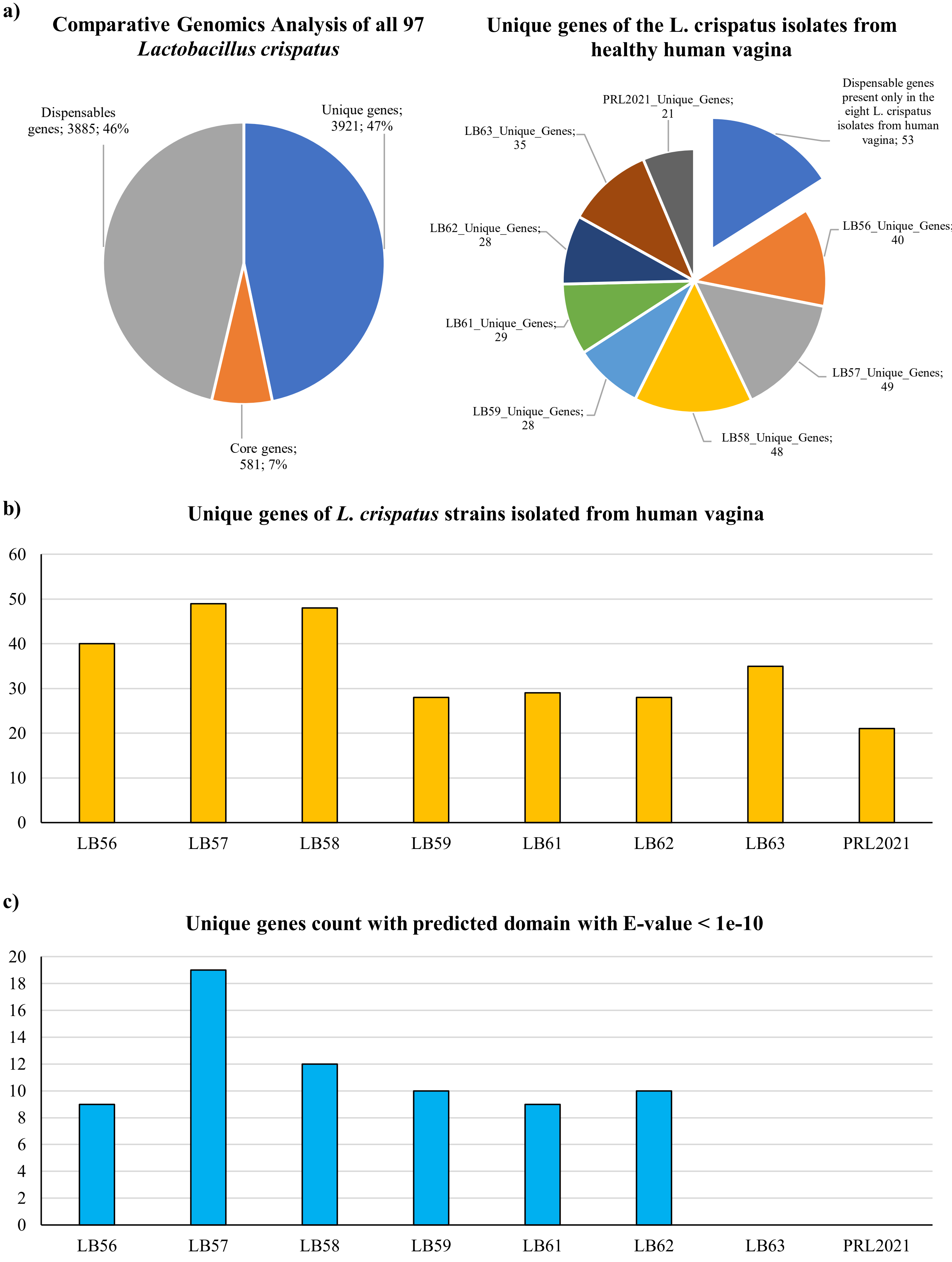
a)
Comparative Genomics Analysis of all 97 Lactobacillus crispatus
Dispensables genes; 3885; 46%
Unique genes; 3921; 47%
Core genes; 581; 7%
Unique genes of the L. crispatus isolates from healthy human vagina
PRL2021_Unique_Genes; 21
LB63_Unique_Genes; 35
Dispensable genes present only in the eight L. crispatus isolates from human vagina; 53
LB62_Unique_Genes; 28
LB56_Unique_Genes; 40
LB61_Unique_Genes; 29
LB57_Unique_Genes; 49
LB59_Unique_Genes; 28
LB58_Unique_Genes; 48
b)
Unique genes of L. crispatus strains isolated from human vagina
60
50
40
30
20
10
0
LB56
LB57
LB58
LB59
LB61
LB62
LB63
PRL2021
c)
Unique genes count with predicted domain with E-value < 1e-10
20
18
16
14
12
10
8
6
4
2
0
LB56
LB57
LB58
LB59
LB61
LB62
LB63
PRL2021

Figure S1
