# Supplementary material for: Probiogenomics Analysis of 97 Lactobacillus crispatus Strains as a Tool for the Identification of Promising Next-Generation Probiotics
Source: Microorganisms. 2020 Dec 30;9(1):73. doi: 10.3390/microorganisms9010073 (PMC7824148; doi:10.3390/microorganisms9010073)
Supplement: Supplementary file 1 [file microorganisms-09-00073-s001.zip › Supplementary Figure legends.pdf]

Supplementary Figure legends

Supplementary Figure 1. The unique gene repertoire of the 97 strains included in the comparative genomics analysis.

Panel A depicts the distribution of unique, disposable and shared genes among the 97 strains, represented through a pie chart. Panel B shows the distribution of unique, disposable and shared genes only among the eight strains isolated from the human vagina sequenced in this study, reported as a pie chart. Panel C reports a bar plot representation of the number of unique genes within each of the eight human strains sequenced in this study. Panel D shows a bar plot representation of the number of unique genes having a functional domain conserved in each of the eight human strains sequenced in this study.

Supplementary Figure 2. Pan-genome and core-genome curves obtained from comparative genomics analysis of the 97 strains included in this study.

Panel A reports the core-genome curve, with the number of shared genes on the y-axis, and the number of genomes included in the analysis on the x-axis. Panel B shows the pan-genome curve, with the number of total genes detected in the genomes of *L. crispatus* reported on the y-axis, and the number of genomes included in the analysis on the x-axis.
