# Supplementary figures and images for: Probiogenomics Analysis of 97 Lactobacillus crispatus Strains as a Tool for the Identification of Promising Next-Generation Probiotics
Source: Microorganisms. 2020 Dec 30;9(1):73. doi: 10.3390/microorganisms9010073 (PMC7824148; doi:10.3390/microorganisms9010073)

a)

Pan-Genome

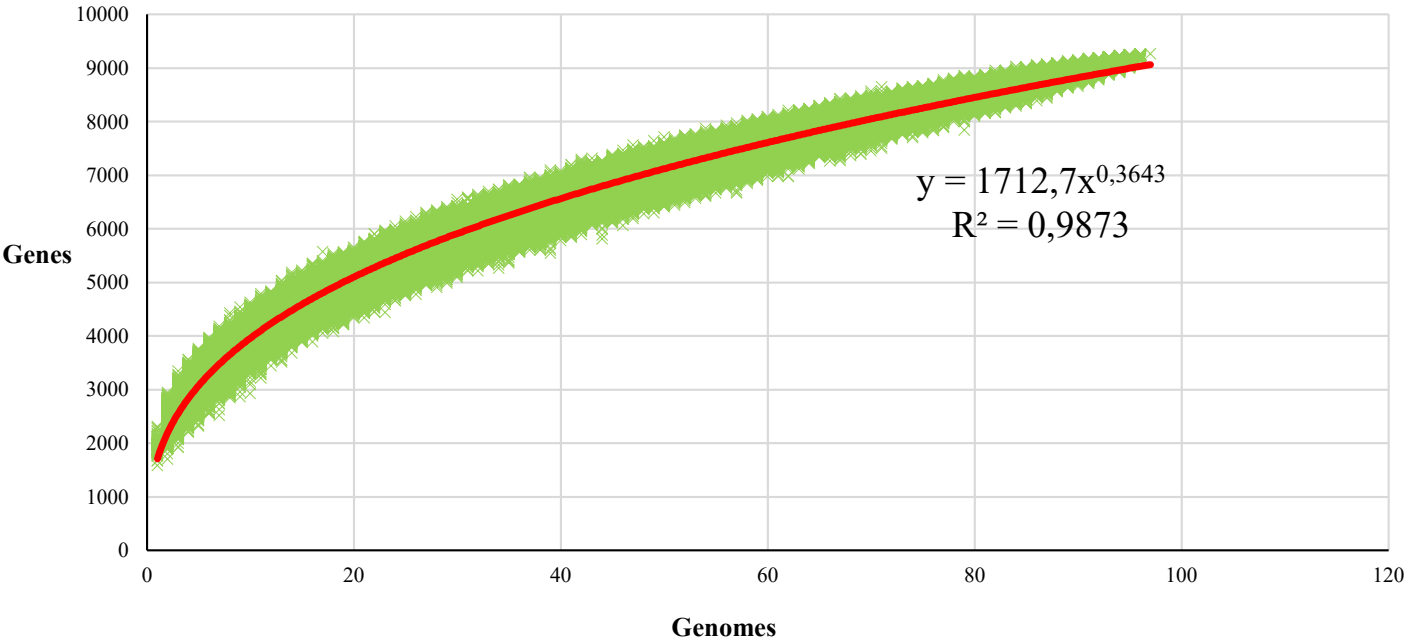

b)

Core-genome

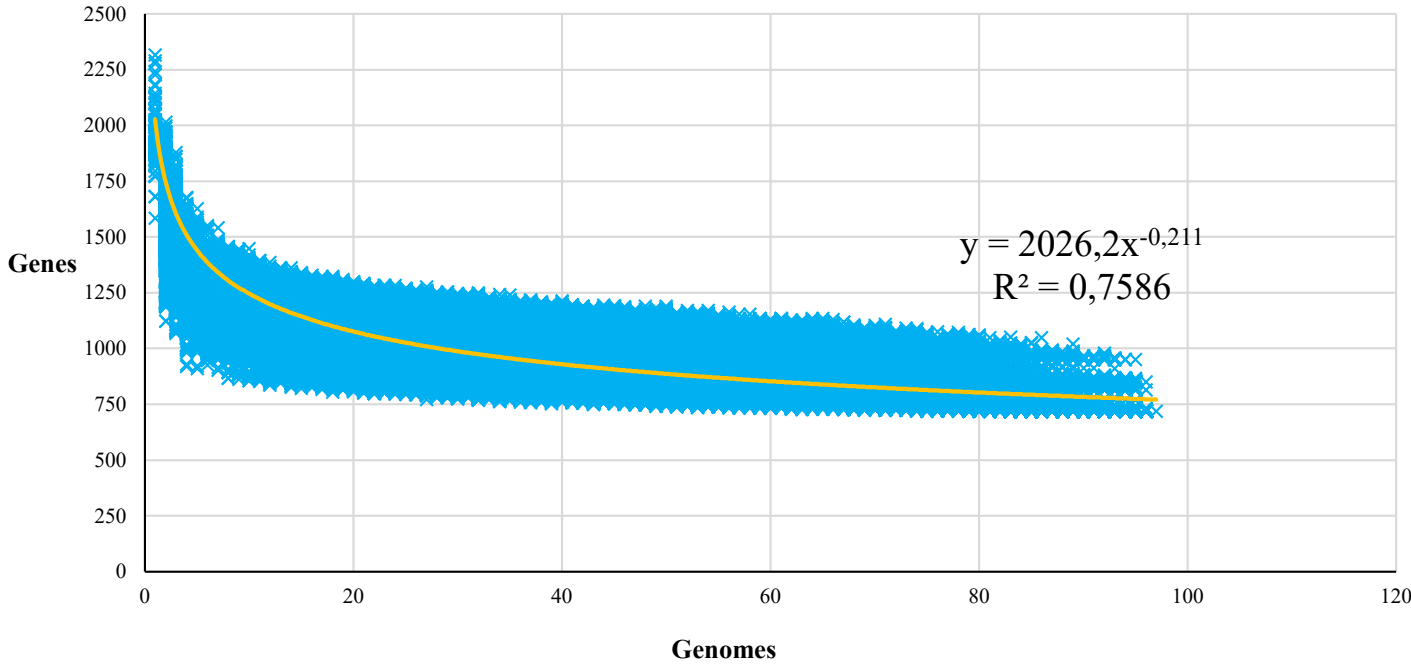

Figure S2

Supplement: Supplementary file 1 [file microorganisms-09-00073-s001.zip › Figure_S2.pdf]
